# Development of the mammalian main olfactory bulb

**DOI:** 10.1242/dev.200210

**Published:** 2022-02-11

**Authors:** Candida Tufo, Subathra Poopalasundaram, Ana Dorrego-Rivas, Marc C. Ford, Anthony Graham, Matthew S. Grubb

**Affiliations:** Centre for Developmental Neurobiology, Institute of Psychiatry, Psychology and Neuroscience, King's College London, London SE1 1UL, UK

**Keywords:** Adult neurogenesis, Maturation, Olfaction, Olfactory bulb development

## Abstract

The mammalian main olfactory bulb is a crucial processing centre for the sense of smell. The olfactory bulb forms early during development and is functional from birth. However, the olfactory system continues to mature and change throughout life as a target of constitutive adult neurogenesis. Our Review synthesises current knowledge of prenatal, postnatal and adult olfactory bulb development, focusing on the maturation, morphology, functions and interactions of its diverse constituent glutamatergic and GABAergic cell types. We highlight not only the great advances in the understanding of olfactory bulb development made in recent years, but also the gaps in our present knowledge that most urgently require addressing.

## Abstract (precis)

**Summary:** This Review describes the morphological and functional maturation of cells in the mammalian main olfactory bulb, from embryonic development to adult neurogenesis.

## Introduction

**Fig. 1.**
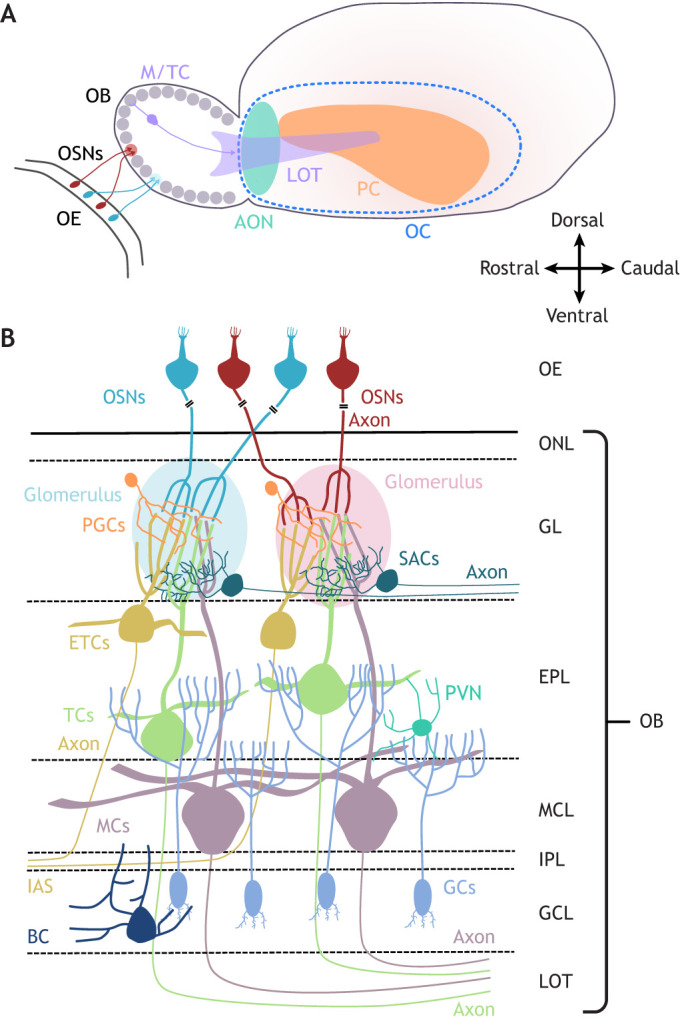
**The mammalian olfactory system and main olfactory bulb circuitry.** (A) Overview of the olfactory system. Olfactory sensory neurons (OSNs) in the olfactory epithelium (OE) of the nose project axons to glomeruli (grey circles) in the olfactory bulb (OB). Mitral and tufted cells (M/TCs) in the OB extend apical dendrites into glomeruli, and send axons through the lateral olfactory tract (LOT) to areas of olfactory cortex (OC), including the anterior olfactory nucleus (AON) and piriform cortex (PC). (B) Simplified diagram of mature OB circuitry. Different colour OSNs represent populations expressing different olfactory receptors, axons of which coalesce into discrete glomeruli. BC, Blanes cell; EPL, external plexiform layer; ETCs, external tufted cells; GCs, granule cells; GCL, granule cell layer; GL; glomerular layer; IAS, intrabulbar association system; IPL, internal plexiform layer; LOT, lateral olfactory tract; MCs, mitral cells; MCL, mitral cell layer; OE, olfactory epithelium; ONL, olfactory nerve layer; PGCs, periglomerular cells; PVN, parvalbumin-expressing EPL neuron; SACs, short-axon cells; TCs, tufted cells.

The mammalian main olfactory bulb (OB) is a highly specialised part of the brain that plays a fundamental role in the sense of smell. It receives its driving input from olfactory sensory neurons (OSNs) in the main olfactory epithelium (OE) of the nose and processes that input through a series of local interactions that involve diverse types of excitatory and inhibitory neurons. The OB then sends the results of this early sensory processing to multiple downstream brain areas collectively referred to as ‘olfactory cortex’ (Klingler, 2017) (Fig. 1A).

OSN inputs to the OB are spatially organised in spherical structures called glomeruli in the outer, glomerular layer (GL) of the bulb (Fig. 1B). OSNs detect airborne odorant stimuli using olfactory receptors (ORs); each OSN expresses just one OR gene from a potential choice of over 1000 (Barnes et al., 2020; Buck and Axel, 1991; Chess et al., 1994; Saraiva et al., 2015). Each mature OB glomerulus receives inputs from OSNs expressing just one type of OR (Treloar et al., 2002; Zou et al., 2004), and all OSNs expressing the same OR type converge their axons onto just one glomerulus (rarely two or three) in both the lateral and medial parts of the OB (Mombaerts et al., 1996; Ressler et al., 1994; Vassar et al., 1994). As OR choice determines both the sensory response properties of OSNs and their spatial targeting in the OB, patterns of glomerular activation represent a combinatorial spatial map of odorant identity (Bozza et al., 2004; Chong et al., 2020; Smear et al., 2013; Soucy et al., 2009). However, activity in OB glomeruli is not simply determined by the properties of their OSN inputs. Instead, it is shaped by complex interactions between the apical dendrites of OB projection neurons, as well as local excitatory and inhibitory GL interneurons (Fig. 1B). Further olfactory processing occurs through reciprocal interactions between the lateral dendrites of bulbar projection neurons and deeper-lying GABAergic granule cells (GCs). Highly processed smell information then leaves the OB for downstream targets via the axons of the lateral olfactory tract (LOT) (Fig. 1).

In mammals, the ability to smell is often crucial from birth (Logan et al., 2012), so the fundamental features of OB circuitry must be present and functional from the earliest postnatal ages. However, this does not preclude a significant degree of postnatal maturation. Indeed, developmental change in the OB persists throughout life, including the continual production of new interneurons via adult neurogenesis. Here, we synthesise the state of the art on OB development from embryo to adult, focusing on the OB's constituent cells and circuits. We do not aim for comprehensive coverage of the myriad genetic and molecular factors involved in OB development; instead, we have highlighted particular genes or proteins where they are the only known molecular contributors to particular processes, or where they appear to be especially important or interesting. Moreover, while acknowledging the huge importance of studying olfactory development in diverse model organisms (Melkman and Sengupta, 2004; Miyasaka et al., 2013; Yang et al., 2019), as well as the wealth of interesting work on alternative olfactory pathways (Katreddi and Forni, 2021), we focus here on the maturation of the main mammalian OB. In practice, this equates to discussing almost exclusively rodent studies and, unless specifically stated, all work described below has been undertaken in mice or rats.

## Early development of the olfactory system

### Initial olfactory nerve development

The olfactory placode, a derivative of the anterior ectoderm, forms first during olfactory system development around embryonic day (E) 9 (all developmental timings refer to mouse) (Treloar et al., 2010). At early stages, a balance of fibroblast growth factor (Fgf) and bone morphogenetic protein (Bmp) signalling is required for the generation of placodal progenitor cells, including cells that contribute to both olfactory and lens placodes (Schlosser, 2014). However, olfactory development is favoured by a shorter period of Bmp signalling; the inhibition of Bmp signalling drives olfactory placode development at the expense of lens, whereas prolonged exposure to Bmp signalling promotes lens formation at the expense of the olfactory placode (Sjödal et al., 2007). The olfactory placode subsequently forms the OE from E10.5, within which OSNs are born. The programme of neurogenesis in the OE involves several transcription factors, including Ascl1, which acts at early stages during the process of OSN generation, and then Neurog1 and NeuroD (Nicolay et al., 2006). Once differentiated, the OSNs project axons that breach the basement membrane of the OE and extend into the subjacent frontonasal mesenchyme. Along with these axons, other cells including gonadotrophin-releasing-hormone-expressing cells also exit the epithelium and collectively form a migratory mass (Valverde et al., 1992). The pioneering olfactory axons project directly towards the ventral telencephalon at ∼E11.5, and then turn sharply towards the rostral telencephalon. After a period of stalling outside of the neural primordium, pioneer axons penetrate at ∼E12, reaching the ventricular zone (Gong and Shipley, 1995).

### Early OB development

The OB develops at the site of entry of OSN axons into the telencephalon. It is unclear how this domain is defined, but it is probably specified as part of forebrain patterning (Kiecker and Lumsden, 2012). Briefly, dorsoventral patterning involves Wnts and Bmps emanating from the dorsal midline and sonic hedgehog (Shh) ventrally, whereas anteroposterior patterning relies on anterior Fgf signalling. Together, these signals establish domains of gene expression that delineate territories within the developing telencephalon. The anterior telencephalon, within which the OB forms, is marked by the expression of ephrin A5 and *Pou3f1* (Hébert et al., 2003).

The development of the OE and the OB are linked. Signals from pioneering OSNs are thought to dampen cell proliferation rates around their site of entry (Gong and Shipley, 1995), increasing neuronal differentiation at the anterior end of the telencephalon and triggering the evagination of the OB. Evagination requires Fgf signalling; conditional deletion of *Fgfr1* in the developing telencephalon prevents OB formation (Hébert et al., 2003). However, this is not because of a failure in the projection of OSN axons to the forebrain nor in any alteration to the patterning of this neural territory. Instead, these animals fail to decrease proliferation rates at the site of the presumptive OB and this region subsequently undergoes abnormal morphogenesis (Hébert et al., 2003). *Fezf1-*deficient mice, which lack a zinc-finger transcriptional repressor, show abnormal axonal projections of OSNs, a reduction in OB size and defects in the organisation of this region, supporting the interdependent development of the OE and OB (Hirata et al., 2006). However, an OB or OB-like structure can form in the absence of OSN innervation of the telencephalon (Jiménez et al., 2000; Levi et al., 2003; Long et al., 2003).

The next phase in OB development is the population of the presumptive bulb by its constituent neurons and its innervation by driving OSN inputs. The maturation of the nose-to-brain projection has been thoroughly covered by many dedicated recent review articles (Imai and Sakano, 2011; Imai et al., 2010; Lodovichi, 2021; Redolfi and Lodovichi, 2021; Sakano, 2020), so we focus on the development of downstream OB circuitry. We also briefly discuss the development of the strong and diverse range of descending (Box 1) and neuromodulatory (Box 2) inputs of the OB, as well as its crucial glial constituents (Table 1).

**Box 1. Development of top-down input to the OB**

Mitral and tufted cells project to a range of olfactory cortical regions (Fig. 1A) that in turn provide strong descending inputs back to olfactory bulb (OB) circuits. These feedback connections are mostly established during the postnatal period (Schwob and Price, 1984; Kostka and Bitzenhofer, 2021). From P1-P2, fibres projecting from the anterior olfactory nucleus (AON) and piriform cortex (PC) start to be visible in the granule cell layer (GCL). Fibres from the PC continue to target the GCL until adulthood, whereas descending projections from the AON/PC border expand into the internal plexiform layer (IPL) at ∼P3–P4 and keep this distribution thereafter. Some fibres from the AON initially innervate the deepest portion of the GCL, expanding to reach the most superficial GCL sublamina by the end of the first postnatal week (Schwob and Price, 1984). A recent study has revealed a clear rostral-to-caudal gradient in the development of top-down input from olfactory cortical areas to the OB: early, perinatally established AON and anterior PC projections are followed in the second postnatal week by the arrival of inputs from more posterior regions of the olfactory cortex (Kostka and Bitzenhofer, 2021).

OB circuits also receive top-down GABAergic input, as well as neuromodulatory cholinergic input (Box 2) from the horizontal limb of the diagonal band (HDB). Adult-born granule cells establish synaptic connections in the GCL with centrifugal fibres from the HDB before they grow dendritic extensions into the external plexiform layer (Whitman and Greer, 2007). Some of these fibres belong to GABAergic neurons in the HDB that project to both the glomerular layer and GCL, with synaptic inputs to immature adult-born GCs that can promote their survival (Hanson et al., 2020).

**Box 2. Summary of developing neuromodulatory input to the olfactory bulb**

The olfactory bulb (OB) receives diverse neuromodulatory inputs (Brunert and Rothermel, 2021), of which some developmental processes are understood.

**Serotonergic.** The OB receives input from serotonergic neurons in the dorsal raphe nuclei (DRN). In rodents and possums, this innervation is postnatal (McLean and Shipley, 1987; Philpot et al., 1994). Fibres of DRN neurons are detected from birth in the caudal OB and later invade rostrally, although the mature innervation pattern remains densest caudally (McLean and Shipley, 1987). DRN fibres innervate the glomerular layer (GL), external plexiform layer (EPL) and internal plexiform layer (IPL). From P0-P14 they selectively increase GL innervation, producing the highest density of serotonergic fibres in the adult OB (McLean and Shipley, 1987).

**Noradrenergic.** Noradrenergic fibres from the locus coeruleus (LC) in the brainstem target different structures in the OB, particularly the granule cell layer (GCL) and IPL (McLean and Shipley, 1991). These fibres are present by P1, become progressively denser throughout development and change orientation. Initially, noradrenergic fibres have a mixed orientation that is both parallel and tangential to the OB surface, before transitioning to a full parallel organisation (McLean and Shipley, 1991). Noradrenergic inputs have at least one exclusively developmental function: from P0-P14, noradrenaline can inhibit granule cells to disinhibit mitral and tufted cell activity (Wilson and Leon, 1988; Pandipati and Schoppa, 2012), which may contribute to early postnatal olfactory discrimination learning (Pandipati and Schoppa, 2012).

**Cholinergic.** The OB mostly receives cholinergic input from horizontal limb of the diagonal band neurons (Box 1), which primarily target the GL and IPL (Le Jeune and Jourdan, 1991). Cholinergic innervation also develops postnatally; between P0-P2, cholinergic fibres innervate the caudal OB and are detected in the GL and (to a lesser extent) in the GCL and the IPL. Cholinergic fibre density subsequently increases and expands into the EPL, establishing the mature innervation pattern by P14 (Le Jeune and Jourdan, 1991).

**Table 1.**
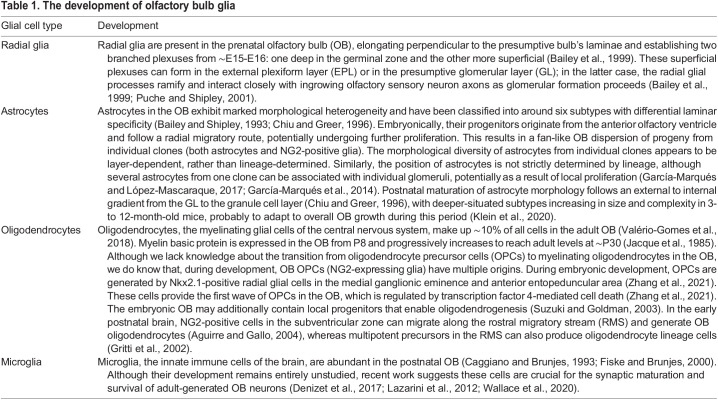
The development of olfactory bulb glia

## OB glutamatergic neuron development

Mitral and tufted cells are the large excitatory projection neurons of the OB, with axons that carry olfactory information to higher processing centres. Morphologically they are alike; both cell types possess an apical dendrite that ends in a dense tuft in the glomerular neuropil and multiple lateral dendrites that extend in the external plexiform layer (EPL) (Fig. 1B). However, they differ in several important aspects (Imamura et al., 2020). Mitral cell somas tend to sit in the deeper mitral cell layer (MCL), whereas tufted cell bodies are sparsely distributed throughout the EPL (Fig. 1B). The axons of these two cell types project to distinct downstream targets (Igarashi et al., 2012) and, functionally, tufted cells fire faster and to lower concentrations of odorants (Burton and Urban, 2014; Fukunaga et al., 2012; Igarashi et al., 2012). The mitral/tufted distinction is almost certainly an oversimplification, with recent transcriptomic studies identifying multiple subtypes of OB projection neurons (Zeppilli et al., 2021). However, most studies of OB development have not distinguished between mitral and tufted neurons, so here we use the term ‘mitral/tufted cells’ (M/TCs) to refer to both cell types together.

A distinct type of OB glutamatergic neuron, although also heterogeneous (Antal et al., 2006; Tatti et al., 2014), is the external tufted cell (ETC). With cell bodies situated in or close to the GL, these neurons have an apical tuft in the glomerular neuropil, with some subtypes also extending lateral dendrites in the EPL (Antal et al., 2006; Pinching and Powell, 1971) (Fig. 1B). They are distinguished from M/TCs by their smaller size, their superficial location and by their axonal projection, which is exclusively intrabulbar (Liu and Shipley, 1994; Schoenfeld et al., 1985).

### Generation and migration

M/TCs are generated from local progenitors in the OB germinal zone, with mitral cells born first (∼E10-E13), followed by tufted cells in an inside-out sequence (∼E13-E18) (Brunjes and Frazier, 1986; Hinds, 1968a; Imamura et al., 2011). ETCs are born later, and can even be generated perinatally, including a minority that originates from the dorsal subventricular zone (SVZ) lining the lateral ventricles (Winpenny et al., 2011) (Table 2).

**Table 2.**
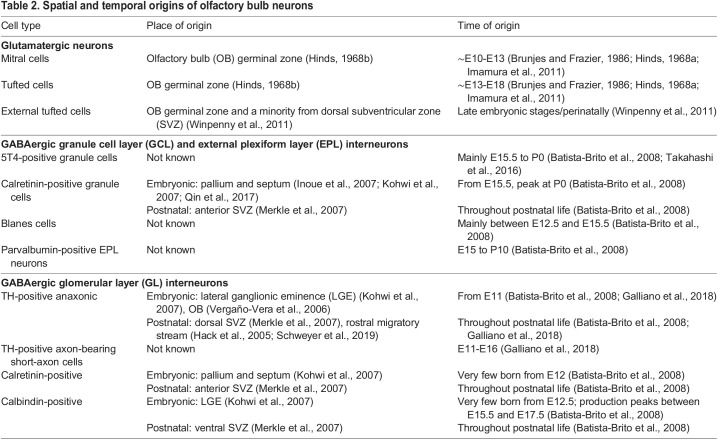
Spatial and temporal origins of olfactory bulb neurons

The molecular basis for the differentiation of M/TCs and ETCs is largely unknown, although transcriptomic screens based on bulk tissue sequencing have identified some developmentally regulated target genes (Campbell et al., 2011; Kawasawa et al., 2016). Fate-mapping experiments have revealed differential contributions to M/TC subpopulations from progenitors expressing transcription factors such as *NeuroD1* (Roybon et al., 2015) and *NeuroG2* (Winpenny et al., 2011). The developmental expression patterns of transcription factors that are exclusive to M/TCs within the OB [such as *Tbr1*, *Tbr2* (also known as *Eomes*) and *Tbet* (*Tbx21*)] have also been mapped over space and time (Nguyen and Imamura, 2019); the early-expressed *Tbr1* is crucial for M/TC development (Bulfone et al., 1998). However, although mature transcriptomic profiles have been mapped for the whole OB (Brann et al., 2020) and for subtypes of OB projection neurons (Zeppilli et al., 2021), we await single-cell transcriptomic analyses applied across M/TC and ETC maturation to build a more coherent picture of the molecular trajectories taken to establish these cell types over development.

After their birth in the OB germinal zone, M/TCs undergo radial migration to the intermediate layer of the presumptive OB, starting at ∼E12 (Blanchart et al., 2006; Hinds, 1968b). Their somas then switch to a tangential orientation and extend long tangential processes with no consistent polarity, before migrating tangentially at ∼E14-E15 to produce an even distribution of M/TCs around the OB (Hinds, 1972). Birth-dating experiments have shown that the earliest-born mitral cells (≤E10) become situated in the dorsal OB, but later-born mitral cells (≥E12) migrate tangentially to the ventral bulb (Imamura and Greer, 2015; Imamura et al., 2011) directed by the adhesion molecule, Tag1 (Cntn2) (Bastakis et al., 2015), and the axon guidance molecule Nrp2 (Inokuchi et al., 2017). The longer migration of ETCs from central OB germinal zones to their final position at the GL-EPL border, however, remains entirely unstudied.

### Morphological development

**Fig. 2.**
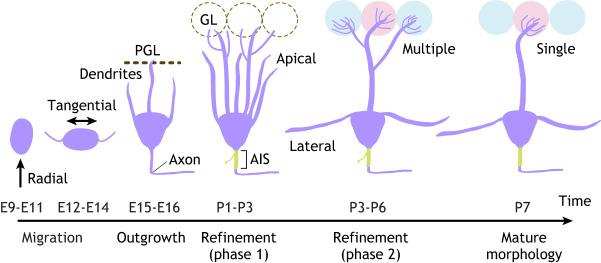
**Morphological development of mitral and tufted cells.** Olfactory bulb glutamatergic projection neurons are born from E9, and undergo phases of radial then tangential migration before extending an axon into the developing lateral olfactory tract and dendrites, which can contact the presumptive glomerular layer (PGL) from E15. Their axon initial segment (AIS) and other proximal axonal features mature by P7. Their apical dendrites ramify within the immature glomerular layer (GL; dotted circles) from P1-P3, then undergo two phases of refinement which produce a mature, highly branched apical tuft within a single mature glomerulus (pink/blue circles) by P7. Mitral and tufted cell lateral dendrites also extend in the first postnatal week.

Here, we discuss the development of M/TCs' distinctive morphology (Fig. 2). The development of ETC dendrites remains completely unstudied, although ETC axons form an exquisitely organised intrabulbar projection, the maturation of which has been well characterised (Box 3).

**Box 3. Development of the intrabulbar association system**

External tufted cells (ETCs) extend an axon from their source glomerulus on one side of the bulb through the internal plexiform layer (IPL) (Fig. 1B) to target postsynaptic granule cells (GCs) directly underneath the homologous glomerulus on the other side of the olfactory bulb (OB), thus directly linking the glomeruli innervated by olfactory sensory neurons (OSNs) expressing the same olfactory receptor. This intrabulbar association system (IAS) is present and appropriately centred beneath the target glomerulus in the early postnatal period, and refines over the first month of life from a broad to a highly focused termination zone (Lorenzon et al., 2015; Marks et al., 2006). IAS pruning is dependent upon OSN-driven activity and is disrupted by sensory deprivation, genetically induced anosmia or reduced OSN spontaneous activity (Lorenzon et al., 2015; Marks et al., 2006). Surprisingly, this activity dependence also extends to IAS maintenance, as the map can be returned to pre-pruning levels of (im)precision by later manipulations that either block sensory input, silence OSNs or inhibit the ongoing production of new-born GCs via adult neurogenesis (Cummings et al., 2014; Lorenzon et al., 2015; Marks et al., 2006). Changes in ETC number alone cannot explain these effects (Marks et al., 2006), but it is still unclear which morphological changes are responsible. Do ETC axon collaterals shrink and regrow and/or reposition themselves even in the late postnatal OB? Imaging this process live in individual ETC axons is required to tackle these crucial questions.

#### Axons

The axon of M/TCs is one of the first identified neurites to extend from the soma during their migration, emerging from ∼E14 and coalescing with neighbouring projection neuron axons to form the early LOT (Blanchart et al., 2006; Hinds, 1972) (Fig. 2). Following the development of these axons as they establish connections with higher olfactory centres is beyond the scope of this Review. However, M/TC axons within the early postnatal OB are distinguished by bleb-like swellings, proximal axonal branches and immature axon initial segments (Blanchart et al., 2006; Hinds and Ruffett, 1973; Malun and Brunjes, 1996).

#### Apical dendrites

Initial growth of M/TC dendrites begins at ∼E14-E16 as the somas of these neurons re-orient radially following their tangential migration and give rise to multiple processes that extend into the presumptive EPL (Blanchart et al., 2006; Hinds, 1972; Santacana et al., 1992) (Fig. 2). M/TC apical dendrites extend into the presumptive GL and contact incoming OSN axon terminals from ∼E16 (Blanchart et al., 2006). Notch ligands released from developing OSNs limit the morphological complexity of M/TC apical dendrites, but not initial migration and neurite outgrowth, as early as E15-E18 (Muroyama et al., 2016). It is unclear why negative regulation is required at this stage, although it may prevent M/TC dendrites from growing out of the developing OB.

After extending multiple processes into different glomeruli, M/TC apical dendrites undergo an extensive process of morphological refinement between postnatal day (P) 0 and P7. By around P6, the vast majority of M/TCs have an extensively branched apical tuft in just one glomerulus (Blanchart et al., 2006; Fujimoto et al., 2019 preprint; Malun and Brunjes, 1996; Santacana et al., 1992; Togashi et al., 2020) (Fig. 2). This refinement process has been split into two distinct phases.

In phase 1 (P1-P3), the number of primary dendrites decreases from around eight to around 4 and densely branched apical tufts grow in most of the remaining processes. This phase occurs independently of neuronal activity (Fujimoto et al., 2019 preprint), but might depend upon physical interactions with OSN axons because early deafferentation (Couper Leo and Brunjes, 2003) or death of OSNs (Kobayakawa et al., 2007; Nishizumi et al., 2019) leads to stunted apical dendrites and a complete lack of tufts.

In phase 2 (P3-P6), one apical dendrite develops a fully ramified tuft in a single glomerulus, with all other apical dendrites pruned away. This extraordinary process of within-neuron developmental refinement requires proper initial dendritic growth because it is permanently disrupted when early branching is perturbed (Muroyama et al., 2016). Pruning also proceeds without any regard for the precise identity of the ‘chosen’ glomerulus. Glomeruli formed from OSNs expressing exogenous ORs contain appropriately pruned, single-targeted M/TC dendrites (Belluscio et al., 2002), and when multiple neighbouring glomeruli are all innervated by OSNs that express the same OR, individual M/TCs form a single apical tuft in just one of those glomeruli (Nishizumi et al., 2019). Moreover, in the absence of dorsally projecting OSNs, dorsal M/TCs close to the ventral OB can establish single apical tufts in ventral glomeruli which they would never normally target (Nishizumi et al., 2019). Finally, there is no relationship between the lineage identity of individual M/TCs and the glomeruli they innervate; neurons derived from the same progenitor never share the same target glomerulus (Sánchez-Guardado and Lois, 2019). It appears that M/TC apical dendrites need to establish contact with just a single glomerulus, but any nearby glomerulus is sufficient.

What dictates the pruning of M/TC apical dendrites? An obvious candidate is neuronal activity; however, neither odour-evoked nor spontaneous activity in OSNs is required for M/TC dendritic refinement, although altering OSN activity can delay the process by a few days (Fujimoto et al., 2019 preprint; Lin et al., 2000; Lorenzon et al., 2015; Matsutani and Yamamoto, 2000). Instead, spontaneous activity in M/TCs appears to be crucial; M/TCs are spontaneously active from birth (Math and Davrainville, 1980), with spontaneous calcium transients in their apical dendrites (Fujimoto et al., 2019 preprint). From P3, these transients are synchronous within glomeruli but asynchronous between glomeruli, providing precisely the kind of patterned activity that might sculpt dendritic pruning. Indeed, lowering spontaneous activity in individual M/TCs leads to permanent pruning deficits, as does disrupting M/TC activity via alterations to glutamatergic signalling (Fujimoto et al., 2019 preprint).

However, spontaneous activity in M/TCs is not the only contributing factor, and olfactory experience can shape dendrite pruning. Repeated exposure to an odorant throughout development increases the number of M/TCs targeting a responsive glomerulus (Liu et al., 2016), and brief enrichment from P2-P4 can accelerate the refinement of M/TC dendrites within a responsive glomerulus (Inoue et al., 2021). It appears that activity levels must be different between the multiple apical dendrites belonging to an individual M/TC; this difference can be entirely provided by spontaneous activity-driven within the M/TC network, but can also be influenced by experience-dependent information driven by OSN inputs. Current data are most consistent with a ‘winner-takes-all’ situation, in which the most active tuft is maintained, with ‘most active’ being defined on a relative, within-cell basis. Evidence for signalling pathways that distinguish ‘to-be-pruned’ versus ‘to-be-stabilised’ dendrites remains a key target for the near future. However, permissive regulators of this process have been recently identified (Aihara et al., 2021), and there is a strong link between OSN-driven dendrite stabilisation and activity-dependent strengthening of OSN-to-M/TC synaptic connections (Inoue et al., 2018, 2021).

#### Lateral dendrites

M/TC lateral dendrites can extend for millimetres in the EPL to make reciprocal dendro-dendritic contacts with GCs. They emerge before birth (Blanchart et al., 2006) and show evidence of extension in the first postnatal week, displaying mature properties from ∼P10 (Imamura and Greer, 2009). Their branching is promoted by the adhesion molecule Nectin1, which is localised in distinct ‘spots’ of putative synaptic contacts with GC dendrites (Fujiwara et al., 2015). Their growth is also decreased in M/TCs with low spontaneous activity (Fujimoto et al., 2019 preprint).

### Functional development

#### Intrinsic properties

Alongside morphological maturation, M/TCs undergo significant functional development in the first postnatal month. Membrane resistance decreases with maturation but resting membrane potential and cell capacitance do not change, suggesting an increase in leak channel density over time without large alterations in total membrane area (Dietz et al., 2011; Duménieu et al., 2015; Yu et al., 2015). Young M/TCs have a larger after-hyperpolarisation associated with a larger calcium influx during repetitive firing, and lower overall firing rate (Duménieu et al., 2015). In addition, developmental narrowing of individual M/TC action potentials, coupled with a decreased sag potential and greater firing irregularity, mean mature M/TCs can better encode high-frequency signals (Yu et al., 2015).

#### Synaptic connections

The early formation of synapses in the OB was characterised in classic electron microscopy studies that assumed axo-dendritic contacts in the presumptive GL were all OSN-to-M/TC connections. Many of these synapses, especially at later developmental stages, could have instead been from OSN terminals onto ETCs (Gire et al., 2012; Hayar et al., 2004). Nevertheless, these axo-dendritic GL synapses were the first formed in the developing OB, appearing as early as E14-E15 in the ventral OB, and undergoing a rapid increase in density from E18 to P6, before peaking at ∼P15-P20 (Blanchart et al., 2008; Hinds and Hinds, 1976). Manipulations of postnatal sensory experience alter the density of both OSN-associated and M/TC-associated presynaptic markers in developing glomeruli, and alter Sema7A expression in OSN terminals (Inoue et al., 2018, 2021). The Sema7A receptor, PlxnC1, is expressed in M/TC dendrites in the first postnatal week only, and specific knockout in M/TCs decreases OSN-to-M/TC synapses (Inoue et al., 2018). OSN firing levels can, therefore, influence synapse formation via activity-dependent Sema7A-PlxnC1 interactions.

Functional features of GL glutamatergic synapses also mature postnatally, with most developmental changes occurring postsynaptically. OSN-evoked responses in ETCs display characteristic features of high presynaptic release probability and presynaptic inhibition from P1 onwards, but undergo significant change up to P28 in postsynaptic receptor composition (Grubb et al., 2008), supporting earlier structural observations that postsynaptic maturation is slower than presynaptic maturation at GL axo-dendritic synapses (Hinds and Hinds, 1976). Glomerular interactions between OB glutamatergic neurons also develop over the first postnatal month; from bidirectional gap-junction electrical coupling at early stages, to unidirectional chemical dendro-dendritic synapses by P30 (De Saint Jan and Westbrook, 2007; Maher et al., 2009). Given the central role of ETCs in mediating OSN-to-M/TC activation in mature circuits (De Saint Jan et al., 2009; Gire et al., 2012), it will be crucial to also understand how this indirect, yet fundamental, excitatory pathway matures over postnatal development.

#### Sensory response properties

How do the above morphological and functional features combine to influence the sensory response properties of maturing OB glutamatergic neurons? For ETCs this is entirely unknown, and even for M/TCs, this is a surprisingly understudied question. Qualitative observations made using either single-unit recordings (Mair and Gesteland, 1982) or labelling for the immediate early gene (IEG) *c-fos* (*Fos*) (Guthrie and Gall, 2003) have shown that M/TCs respond to odorants from birth with different temporal spiking patterns and spatial odorant-evoked activation patterns from those of mature projection neurons. Quantitative analysis of single-unit recordings has found that mature M/TCs fire more spikes to odorant stimuli and display tightly time-locked responses, but surprisingly are no different in their selectivity to a range of chemically related stimuli than their immature counterparts (Fletcher et al., 2005). Whether odorant-response properties undergo significant developmental changes when assessed with modern *in vivo* functional approaches, especially during the process of M/TC dendritic pruning in the first postnatal week, remains an urgent outstanding question.

## OB GABAergic interneuron development

GABA-releasing interneurons greatly outnumber glutamatergic neurons in mature OB circuits (Shepherd et al., 2004). Some bulbar GABAergic neurons are generated during embryonic development, but most are generated during the first weeks of the postnatal period and continue to be produced throughout life via adult neurogenesis (Lledo et al., 2008), a process that may also generate a small proportion of glutamatergic interneurons (Brill et al., 2009). OB GABAergic interneurons are mainly classified into two broad populations based on their laminar location: GCs, with somas mainly located in the granule cell layer (GCL), and juxtaglomerular GABAergic neurons in the GL (Fig. 1B). Other OB GABAergic interneuron types include Blanes cells, which specifically inhibit GCs in the GCL (Pressler and Strowbridge, 2006), and parvalbumin-expressing EPL neurons, which provide broadly tuned inhibitory input to M/TCs (Miyamichi et al., 2013) (Fig. 1B). However, as their development remains almost completely uncharacterised (Batista-Brito et al., 2008), we focus here on GC and GL interneuron maturation.

GCs are the most numerous interneuron type in the OB. They lack an axon and release GABA from spiny apical dendrites that extend into the EPL to interact reciprocally with the lateral dendrites of M/TCs (Burton, 2017). The majority of juxtaglomerular GABAergic neurons are also anaxonic (Galliano et al., 2018; Kosaka and Kosaka, 2011), and these axonless GL interneurons are collectively referred to as periglomerular cells (PGCs). They have small somas and spatially limited dendritic arbours that release GABA (and sometimes dopamine) to influence local glomerular activity (Burton, 2017). The second major subtype of juxtaglomerular GABAergic neurons are ‘short-axon cells’; these have larger somas, broader glomerular dendritic arbours and, paradoxically, a long axon that makes distant interglomerular projections (Galliano et al., 2018; Kiyokage et al., 2010; Kosaka and Kosaka, 2011; Pinching and Powell, 1971) (Fig. 1B).

Both GCs and juxtaglomerular GABAergic interneurons are highly heterogeneous and can be further classified into different subgroups with distinct neurochemical, morphological and functional characteristics (Kosaka et al., 1997, 1998; Nagayama et al., 2014; Parrish-Aungst et al., 2007). Based on the location of their dendritic extension in the EPL, GCs can be classed as ‘superficial’, ‘intermediate’ or ‘deep’ (Greer, 1987; Mori et al., 1983; Takahashi et al., 2018). Biochemical distinctions can also be made between GCs that express either the oncofoetal trophoblast glycoprotein, 5T4 (Tpbg) or calretinin (Calb2) (Batista-Brito et al., 2008; Imamura et al., 2006; Takahashi et al., 2018).

In mice, GL GABAergic neurons are divided into mutually exclusive calbindin-, calretinin- and tyrosine hydroxylase (TH; the rate-limiting enzyme for dopamine synthesis)-expressing subclasses (Kosaka et al., 1995; Parrish-Aungst et al., 2007; Toida, 2008). Although anaxonic PGCs can be any of these biochemical subtypes, current evidence suggests that GL short-axon cells are exclusively TH-positive (Galliano et al., 2018; Kiyokage et al., 2010; Kosaka and Kosaka, 2008). There is also evidence for a nitric oxide synthase-expressing subset of GL interneurons (Crespo et al., 2003), the development of which remains almost completely uncharacterised.

The nomenclature and classification of OB GABAergic neuron subtypes can be both confusing and contentious, so we have endeavoured to provide an evidence-based framework that is at least clear and developmentally relevant. However, it is unlikely to be definitive; indeed, there is increasing evidence for a high degree of heterogeneity within each OB interneuron subtype (Galliano et al., 2018, 2021; Kosaka and Kosaka, 2011, 2016; Parrish-Aungst et al., 2007; Takahashi et al., 2018).

### Temporal and spatial origins

The heterogeneity of OB GABAergic neurons is largely determined by a spatial-temporal transcriptional code of their site of origin. During embryonic development, OB interneurons originate from the ventral telencephalon (subpallium) (Southwell et al., 2014) comprising the medial, lateral and caudal ganglionic eminences (MGE, LGE and CGE, respectively) and the preoptic/anterior entopeduncular area (Turrero García and Harwell, 2017). Embryonic neurogenesis mostly starts at E10, after neuroepithelial cells located in the ventricular zone (VZ) that line the walls of the lateral ventricles differentiate into radial glia. During this process, neuroepithelial cells downregulate some epithelial features and start expressing astroglial markers. As development proceeds, the SVZ is formed from the proliferation of radial glia at basal VZ locations, and becomes the main proliferative region between E13 and E14 (Götz and Huttner, 2005; Turrero García and Harwell, 2017). The earliest cohort of OB interneurons is generated between E12.5 and E14.5, primarily from the LGE (Batista-Brito et al., 2008; Kohwi et al., 2007; Tucker et al., 2006; Wichterle et al., 1999, 2001) (Table 2). Specifically, the dorsal LGE is populated by distinct progenitors expressing *Dlx2*, *Gsh2* (*Gsx2*) and *Er81* (*Etv1*) that generate all major OB interneuron subtypes (Qin et al., 2017; Stenman et al., 2003; Wichterle et al., 2001). Mutation of these and other transcription factors (e.g. *Arx* or *Sp8*) results in strongly reduced numbers of GABAergic interneurons in both the GCL and GL (Guo et al., 2019; Li et al., 2018; Stenman et al., 2003; Waclaw et al., 2006; Yoshihara et al., 2005; Yun et al., 2003).

Lineage tracing of Dlx1/2 precursors, which give rise to most OB GABAergic interneurons, has revealed that the first OB interneurons to be conspicuously generated from E12.5 are the TH-expressing, dual dopamine- and GABA-releasing cells (Batista-Brito et al., 2008). This early-born population includes the distinct subset of axon-bearing OB dopaminergic neurons that, unlike their anaxonic TH-expressing counterparts, are exclusively generated in early embryonic development (Galliano et al., 2018) (Table 2). As development proceeds, the production of TH-positive neurons decreases, and the production of calbindin-positive and calretinin-positive cells progressively increases. Although some embryonic-derived progenitors give rise to OB interneurons expressing calretinin, most calretinin-positive cells are generated after birth (Batista-Brito et al., 2008). Interestingly, grafting embryonic progenitors from the LGE into the adult brain results in the production of both TH-positive and calbindin-positive interneurons, but not calretinin-positive cells (Kohwi et al., 2007), suggesting that the few calretinin-expressing cells derived from embryonic precursors might be generated in non-LGE locations.

Outside the LGE, other embryonic sources of OB interneurons are the pallium and the septum, which mainly give rise to calretinin-expressing cells (Inoue et al., 2007; Kohwi et al., 2007; Qin et al., 2017). Pallial and septal progenitors expressing *Emx1* and *Dlx5/6* produce a subset of OB interneurons that includes calretinin-positive cells (Kohwi et al., 2007) (Table 2). Septum-derived Zic1/3 progenitors are required for a different OB interneuron subset, including some TH-positive cells (Inoue et al., 2007), although more recent genetic fate-mapping experiments suggest that medial septal progenitors mainly give rise to calretinin-positive interneurons, with minimal contribution to other OB cell types (Qin et al., 2017).

Endogenous embryonic OB precursor cells also contribute to the generation of GABAergic OB interneurons (Vergaño-Vera et al., 2006), at least until early postnatal life (Lemasson et al., 2005). OB transplants and dissociated cultures of precursors taken from the E13.5 OB can differentiate into GABAergic and dopaminergic cells (Vergaño-Vera et al., 2006) (Table 2), although it is unclear whether these locally born cells comprise distinct interneuron subpopulations.

**Fig. 3.**
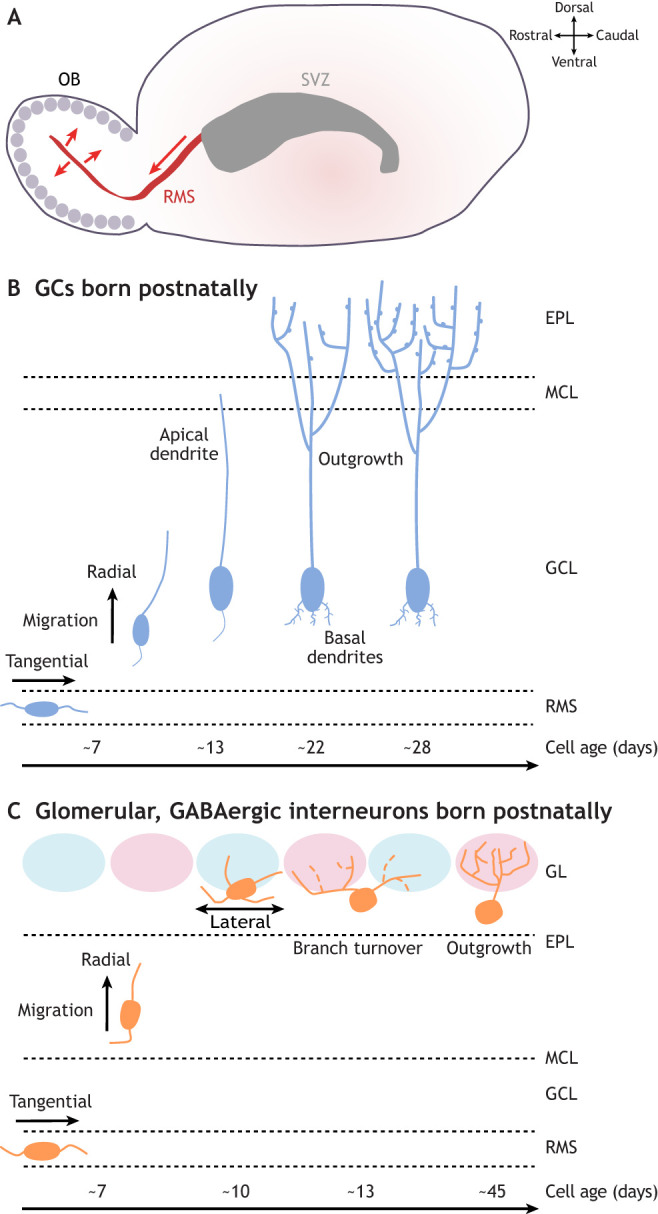
**Morphological maturation of postnatally generated olfactory bulb granule cells and glomerular layer GABAergic interneurons.** (A) Olfactory bulb (OB) neurons born postnatally are generated in the subventricular zone (SVZ) lining the lateral ventricle, then migrate tangentially along the rostral migratory stream (RMS) to reach the OB, before migrating radially out of the RMS to integrate into the granule cell layer (GCL) and glomerular layer (GL). (B) Morphological maturation of granule cells (GCs) generated postnatally. After migrating tangentially in the RMS, immature neurons migrate radially into the GCL, before extending an apical dendrite to the mitral cell layer (MCL). Over the next ∼2 weeks, the apical dendrites extend further into the external plexiform layer (EPL), branch extensively and gain spines. Smaller basal dendrites also develop over this time period. (C) Morphological maturation of GL GABAergic interneurons generated postnatally. These cells also undergo tangential migration in the RMS, then radial migration up to the GL, where they continue to move laterally between glomeruli for some time. At ∼2 weeks of cell age, they then experience a period of branch turnover with dynamic additions and retractions, before ramifying extensively, usually within a single glomerulus, by the time they are ∼6 weeks old.

Neurogenesis of OB interneurons continues after birth. OB interneuron generation reaches its peak in the first few postnatal weeks (Batista-Brito et al., 2008) and continues, albeit at a steadily decreasing rate, throughout adult life (Conover and Todd, 2017; Lledo et al., 2008). Neurogenic capacity is retained during postnatal development and adulthood in the SVZ, a germinal zone lining the walls of the lateral ventricles (Alvarez-Buylla and Garcia-Verdugo, 2002; Obernier and Alvarez-Buylla, 2019; Tramontin et al., 2003) (Fig. 3A). Here, slowly dividing type-B astrocytes function as primary precursor cells, giving rise to rapidly dividing transit-amplifying cells (type-C). Type-C cells, in turn, generate migrating neuroblasts (type-A cells) that make their way towards the OB (Doetsch et al., 1999). Cre-lox fate mapping of telencephalic neuroepithelium has revealed that the postnatal SVZ is populated by a heterogeneous pool of stem cells, derived from the MGE, LGE and embryonic cortex, which remain quiescent until activated in adulthood (Fuentealba et al., 2015; Young et al., 2007). LGE- and cortex-derived progenitors give rise to distinct types of OB interneurons: cortex-derived progenitors produce the majority of calretinin-positive neurons but no calbindin-positive cells, whereas LGE-derived progenitors mainly give rise to calbindin-positive interneurons. TH neurons are generated from both cortex-derived and LGE-derived progenitors (Young et al., 2007).

There is also considerable regional diversity within the SVZ (Chaker et al., 2016). Dorsal SVZ regions mainly produce TH-positive cells and superficial GCs, ventral regions give rise to calbindin-positive neurons and deep GCs, and anterior regions produce both glomerular and granule calretinin-positive cells, in a location-dependent code that is largely maintained in the adult SVZ (Merkle et al., 2007; Paul et al., 2017) (Table 2). Lineage-tracing studies have shown that both early postnatal and adult SVZ progenitors produce clones that are usually restricted to either GC or GL interneuron types, although mixed clones can occur (Figueres-Oñate et al., 2019; Fuentealba et al., 2015). This suggests a large degree of fate specification of OB interneurons at their SVZ site of origin. There is also temporal dependence here: superficial GCs are preferentially generated, in a *Pax6*-dependent manner (Kohwi et al., 2007), from the dorsal SVZ from embryonic development until early postnatal life, whereas deeper GCs are generated from the ventral SVZ in late postnatal periods and throughout adulthood (Hinds, 1968a; Lemasson et al., 2005; Merkle et al., 2007). Accordingly, genetic ablation of new-born neurons in adults results in a lack of deep, but not superficial, GCs (Imayoshi et al., 2008). Early-born GCs also have a higher probability of 5T4 versus calretinin expression (Batista-Brito et al., 2008; Takahashi et al., 2016).

Finally, progenitor cells are also present in the rostral migratory stream (RMS) throughout life (see below), where they give rise to OB interneurons, especially TH-expressing cells (Alonso et al., 2008; Gritti et al., 2002; Hack et al., 2005; Schweyer et al., 2019).

### Migration to the OB

SVZ-born cells migrate long distances before reaching the OB (Lois and Alvarez-Buylla, 1994). During embryonic development, small cells with fusiform cell bodies and growth cone extensions migrate from the rostral region of the LGE through a presumptive RMS towards the developing OB (Tucker et al., 2006). The RMS anlage forms between E15 and E17, slightly after the emergence of the ganglionic eminence but before the bulging of the OB (Pencea and Luskin, 2003). The RMS contains neuroblasts migrating in homotypic chains (Lois et al., 1996; Pencea and Luskin, 2003), and extends from the anterior region of the lateral ventricle (deriving from the dorsal LGE) towards the OB. It terminates in the subependymal layer, the central region of the GCL (Pencea and Luskin, 2003; Sun et al., 2010).

In the adult RMS (Fig. 3A), chains of neuroblasts are ensheathed by specialised astrocytes, forming glial tubes (Lois et al., 1996; Peretto et al., 1997), which regulate their migration (Alvarez-Buylla and Garcia-Verdugo, 2002; Sun et al., 2010). The speed and direction of neuroblast migration are regulated by cell-to-cell adhesion and extracellular matrix molecules, chemoattractive and chemorepulsive secretory factors, and local signals from blood vessels (Sun et al., 2010). In addition, polysialylated neural cell adhesion molecules (NCAMs) are highly expressed in migrating neuroblasts and are important for perinatal migration before glial tubes are formed (Cremer et al., 1994; Hu et al., 1996; Law et al., 1999; Ono et al., 1994). Neuronal migration from the SVZ to the OB takes as little as 3-6 days (James et al., 2011; Lois and Alvarez-Buylla, 1994; Luskin, 1993) and accelerates as development proceeds (Lemasson et al., 2005).

RMS migration ends when neuroblasts detach from their migrating chains and start to move radially into the OB. This transition requires the cell-adhesion molecule, tenascin-R, in adult neurogenesis but not during development (David et al., 2013; Saghatelyan et al., 2004). In the OB, most neuroblasts terminate their migration in the GCL and differentiate into GCs. A smaller percentage migrate radially towards, and then laterally within, the GL, where they differentiate into PGCs (Liang et al., 2016). The molecular and cellular factors underlying this migratory choice of laminar targets remain almost entirely unknown (Bartolini et al., 2013).

### GC morphological development

GCs are present in the GCL in early postnatal life. As they mature, they enlarge their soma, elongate and ramify their leading apical process in the EPL, and extend their basal dendritic processes deeper in the GCL (Kishi, 1987). In 5T4-expressing GCs, but not other types, the development of apical dendritic complexity is regulated by 5T4 levels (Takahashi et al., 2016).

The morphological maturation of postnatally born GCs follows the same general pattern but is understood in far more detail (Fig. 3B) (Carleton et al., 2003; Mizrahi, 2007; Petreanu and Alvarez-Buylla, 2002; Whitman and Greer, 2007). Radially migrating immature GCs that are ∼7 days old (all timings in this section refer to cell age) elongate a prominent leading process and a small trailing process. From day 9-13, an unbranched dendrite extends towards – but not beyond – the MCL. From day 11 to day 22, this apical dendrite reaches the EPL and begins to branch (Petreanu and Alvarez-Buylla, 2002) and, from day 14, spines start to form (Whitman and Greer, 2007). These later processes of dendritic growth and spine formation are regulated by olfactory experience because both are negatively impacted by prolonged sensory deprivation via unilateral naris (i.e. nostril) occlusion (UNO) (Saghatelyan et al., 2005). Together with the establishment of reciprocal dendro-dendritic synapses, spine density on GC apical dendrites gradually increases until day 28 of their maturation, then plateaus, before decreasing by day 56 (Whitman and Greer, 2007).

After this period of morphological development and spine formation, about half of newly generated adult-born GCs die in an activity-dependent manner, possibly to remove surplus neurons that have not appropriately integrated into the circuit (Lemasson et al., 2005; Lin et al., 2010; Magavi et al., 2005; Petreanu and Alvarez-Buylla, 2002; Rochefort et al., 2002). However, recent evidence suggests adult-born cell death under baseline conditions may be an artefact of specific birth-dating methods (Platel et al., 2019). Those same birth-dating methods have revealed much higher survival rates in perinatally born GCs (Lemasson et al., 2005), but it is currently unclear how these early-generated cells are protected, or why.

### GC functional development

#### Intrinsic properties

As early as P2-P4, and before they start receiving significant synaptic input, most neonatal GCs have large voltage-dependent sodium currents and are capable of firing action potentials (Carleton et al., 2003). From P5 onwards, GCs show no change in membrane resistance, but their resting membrane potential hyperpolarises and their first-spike latency increases with age, suggestive of an overall decrease in excitability with maturation (Dietz et al., 2011). Conversely, in adult-born GCs, spike firing is one of the last features to emerge (Carleton et al., 2003). Immature adult-born GCs rest at depolarised membrane potentials and have relatively high input resistance; these properties gradually hyperpolarise and decrease, respectively, with maturation (Carleton et al., 2003). Immature adult-generated GCs have voltage-dependent potassium currents but only very small voltage-dependent sodium currents, which increase with maturation until action potential generation is finally possible when cells are around 2 weeks old, long after synaptic inputs have been established (Carleton et al., 2003; Panzanelli et al., 2009). Further studies are needed to investigate whether distinct GC subtypes display differences in this process.

#### Synaptic connections

GC dendritic spines contact the lateral dendrites of M/TCs in the EPL, forming reciprocal excitatory-inhibitory dendro-dendritic synapses, which are a key feature of mature OB circuitry. In early development, the first unequivocal dendro-dendritic synapse in the EPL was detected with electron microscopy at E18. M/TC-to-GC excitatory connections generally preceded GC-to-M/TC inhibitory synapses, with fully reciprocal dendro-dendritic synapses not apparent before P1 (Hinds and Hinds, 1976). The neurexin ligand cerebellin (Cbln1), which inhibits the interactions of signalling molecules shared by pre- and post-synaptic specialisations on mitral cells, is crucial for the organisation of these dendro-dendritic contacts (Wang et al., 2021). Functionally, the reciprocal dendro-dendritic inhibition experienced by M/TCs due to GC-mediated feedback is present at P3 and increases in amplitude until P15 before decreasing again by P30 (Dietz et al., 2011). These changes are likely mediated by a number of developmental alterations, including changes in synapse number and GC intrinsic excitability (Dietz et al., 2011; Whitman and Greer, 2007).

Adult-generated OB neurons express functional synaptic receptors even while they are migrating in the RMS and start to receive functional GABAergic, and then glutamatergic inputs, soon after they arrive in the OB (Carleton et al., 2003; Panzanelli et al., 2009). As GCs mature and make spines, the frequency and amplitude of excitatory inputs increase significantly (Carleton et al., 2003), possibly correlating with the formation of dendro-dendritic synapses between mitral and GCs. Here, again, adult-born GC inputs precede their outputs; inhibition of M/TCs from adult-generated GCs takes longer (∼6 weeks) to mature than even late-developing GC spiking ability (∼2 weeks) (Bardy et al., 2010). Indeed, this process appears to be significantly delayed in adult-born versus embryonically generated GCs. Although input and output synaptic specialisations form close in time in early-born GCs, the maturation of output structures significantly lags that of input structure in adult-born cells (Kelsch et al., 2008; Whitman and Greer, 2007). The mature synaptic features of GCs can also differ between developmentally and adult-generated cells. For example, early-born superficial GCs and late-born deep GCs preferentially contact the lateral dendrites of tufted and mitral cells, respectively (Geramita et al., 2016; Lemasson et al., 2005; Orona et al., 1983). Within one GC subtype, however, fully mature early- and late-born calretinin-positive GCs have indistinguishable morpho-functional properties (Hardy et al., 2018).

GCs also receive glutamatergic synaptic input onto their soma and basal dendrites in the GCL, from axon collaterals of M/TCs or from descending projections from higher olfactory processing regions (Kishi et al., 1984; Luskin and Price, 1983) (Box 1). These are structurally evident when adult-born cells are 10 days old (Whitman and Greer, 2007), and are a notable site for age-dependent synaptic plasticity; basal inputs onto immature but not mature adult-generated GCs can undergo long-term potentiation after theta burst stimulation (Nissant et al., 2009).

#### Sensory response properties

For such a numerous and crucial cell type in OB circuit function, very little is known about the development of sensory response properties in GCs in general, let alone the maturation of distinct response characteristics in different GC subtypes (Malvaut et al., 2017; Hardy et al., 2018). IEG labelling has found that sparse subpopulations of neonatal GCs, especially deep GCs, are activated by odorant exposure; during development, the distribution of GCs activated by a given odorant becomes more continuous within broad zones of the GCL (Guthrie and Gall, 2003). In adult-generated GCs, IEG labelling has shown that immature cells respond more strongly to novel odours, and show distinct plastic changes in response to repeated odorant presentation (Magavi et al., 2005). Recent functional imaging work has suggested that odorant tuning might become broader with maturation in adult-born GCs (Quast et al., 2017); however, subsequent reassessment of this question using more accurate labelling and longitudinal calcium imaging has shown that, for the most part, GC responses become weaker but more selective as adult-generated cells mature (Wallace et al., 2017). Moreover, responses of adult-born GCs grow in amplitude for relevant stimuli during the acquisition of a difficult sensory discrimination task (Wu et al., 2020). Immature adult-born GCs, therefore, have distinct intrinsic, synaptic, plastic and sensory response characteristics, all of which may enable them to contribute to unique behavioural demands.

### Morphological development of GABAergic glomerular interneurons

Glomerular interneurons start populating the more superficial portion of the OB before the GL is fully formed, and are present in the GL from birth (Bastianelli and Pochet, 1995; McLean and Shipley, 1988; Saino-Saito et al., 2004). In rabbits, distinct subtypes of GL GABAergic cells are evident in early postnatal life, including neurons morphologically similar to anaxonic PGCs and larger short-axon cells (Bufler et al., 1992). Dopaminergic, TH-expressing GL interneurons are present in both major subtypes from birth, with small anaxonic cells and larger axon-bearing neurons present at this age (Galliano et al., 2018; McLean and Shipley, 1988). Owing to their exclusively embryonic origins, larger axon-bearing dopaminergic neurons become proportionally rarer as new smaller anaxonic TH-positive cells are added to the GL postnatally (Galliano et al., 2018; McLean and Shipley, 1988). Despite their later origin compared with TH-positive neurons (Batista-Brito et al., 2008), both calretinin-positive and calbindin-positive glomerular cells are present perinatally and continue to expand in number until ∼P20 (Bastianelli and Pochet, 1995). However, it is not known how any of these GL cell types mature morphologically over this early developmental period.

The morphological development of adult-born GL interneurons is better understood (Fig. 3C). Immature adult-generated juxtaglomerular neurons (∼9-10 days old; timings in this section refer to cell age) are still migrating laterally within the GL but have already formed substantial dendrites, which extend into the glomerular neuropil (Kovalchuk et al., 2015; Liang et al., 2016; Mizrahi, 2007; Su et al., 2020 preprint). At around day 9-13, adult-born GL neurons develop a highly dynamic dendritic tree with continuous branch additions and retractions (Livneh et al., 2009; Mizrahi, 2007; Su et al., 2020 preprint). This early remodelling is a particular feature of non-spiny neurons, whereas spiny adult-born cells have relatively stable dendrites with highly dynamic spines (Mizrahi, 2007). With maturation, the dendritic tree gradually increases in complexity up to ∼45 days (Livneh and Mizrahi, 2011a; Livneh et al., 2009, 2014; Mizrahi, 2007), although a decrease in dendritic complexity has also been observed ∼13-45 days (Su et al., 2020 preprint), perhaps because of approaches that differentially target distinct interneuron subtypes. Dendritic development in adult-born GL neurons is regulated in an activity-dependent manner, as odour enrichment accelerates the morphological maturation of these cells in enriched loci (Livneh et al., 2009). When mature, adult-born GL neurons acquire another form of activity-dependent plasticity, whereby odour enrichment stabilises synaptic connections (Livneh and Mizrahi, 2011b). Sensory experience can also regulate the survival of adult-born GL neurons (Alonso et al., 2006), although these effects are less consistent than for adult-generated GCs (Mouret et al., 2008; Saghatelyan et al., 2005). Further studies are needed to elucidate how morphology and survival are regulated in distinct subtypes of adult-born GL GABAergic neurons.

### Functional development of GABAergic GL interneurons

#### Intrinsic properties

Electrophysiological recordings in broadly-identified GL interneuron populations have identified key developmental features in the early postnatal period but also – unsurprisingly for such a diverse group of cell types – a great deal of heterogeneity. Early-generated GL interneurons decrease their membrane resistance with age (Grubb et al., 2008) and show a general trend for larger voltage-dependent sodium currents and greater spike firing capacity over developmental time, reaching mature properties by ∼P30 (Belluzzi et al., 2003; Grubb et al., 2008; McQuiston and Katz, 2001; Puopolo and Belluzzi, 1996, 1998; Senseman, 1996). Postnatally born PGCs show similar trends and increase their membrane capacitance over time (Belluzzi et al., 2003; Grubb et al., 2008), consistent with the overall morphological growth of these cells. Notably, unlike adult-born GCs, adult-born PGCs can fire spikes at very early maturational stages (Bardy et al., 2010; Carleton et al., 2003; Grubb et al., 2008).

Given the heterogeneity observed in the general GL GABAergic population, it is crucial to know how functional development proceeds in individual cell subclasses. However, little is understood here. Calretinin-expressing neurons are unusual in not ever fully maturing; instead, they possess ‘immature-like’ weakly excitable properties at all ages (Benito et al., 2018; Fogli Iseppe et al., 2016). Postnatally generated dopaminergic neurons express *Th* mRNA, but not TH protein, while they are still migrating towards the GL (Pignatelli et al., 2009; Saino-Saito et al., 2004). Using this feature to identify putative young dopaminergic cells in the MCL/IPL has shown that they have immature voltage-gated conductance profiles and a depolarised chloride reversal potential (Pignatelli et al., 2009). However, whether *Th* mRNA-expressing cells in the deeper layers of the OB eventually migrate and become mature dopaminergic neurons has not been directly shown.

#### Synaptic properties

Structurally, the first dendro-dendritic synapse between a presumed juxtaglomerular neuron and an M/TC apical dendrite was observed at E15 (Hinds and Hinds, 1976), an age at which the juxtaglomerular cell involved must have been either locally generated or not GABAergic. Such structural GL dendro-dendritic synapses develop with a slight delay compared with axo-dendritic synapses involving olfactory axons, increasing postnatally alongside increasing GL interneuron generation (Batista-Brito et al., 2008) to peak at ∼P15-P20 (Hinds and Hinds, 1976). In line with this structural maturation, young neonatal GL interneurons receive functional spontaneous glutamatergic and GABAergic inputs that increase in frequency throughout the first postnatal month (Grubb et al., 2008; Puopolo and Belluzzi, 1996). GL interneurons can also receive monosynaptic input from OSN axons as early as P1, and undergo subsequent postsynaptic development of these connections involving different contributions from distinct glutamatergic receptor types (Grubb et al., 2008).

Similar features of synaptic maturation have been observed in adult-generated GABAergic glomerular cells. Structurally, overexpression of PSD95-GFP has revealed a maturational increase in glutamatergic postsynaptic sites that plateaus after cells are ∼6 weeks of age (Livneh and Mizrahi, 2011a). Live imaging of this probe *in vivo* has shown that synaptic sites in adult-born PGCs are highly dynamic at all stages, but especially when cells are immature and establish contacts in glomerular networks (Livneh et al., 2009). Functionally, adult-born PGC maturation is extremely similar to that of neonatal GL GABAergic cells, with increasing frequencies of spontaneous inputs and alterations in OSN-driven postsynaptic properties (Belluzzi et al., 2003; Grubb et al., 2008).

#### Sensory response properties

In development, IEG labelling has found odour-specific activity at birth in scattered juxtaglomerular neurons. With later maturation, the density of these c-fos-labelled neurons increases, correlating with the increasing number of glomerular interneurons generated postnatally (Guthrie and Gall, 2003). We know more about the development of sensory response properties in adult-born glomerular interneurons, which can respond to relevant sensory stimuli with calcium transients ∼48 h after they arrive in the GL at ∼9 days old. This early odour responsiveness is similar to that of neighbouring mature GL cells (Kovalchuk et al., 2015). However, electrophysiological recordings show that immature adult-born glomerular interneurons have higher odorant responsiveness, but lower odour selectivity compared with their mature counterparts (Livneh et al., 2014), a similar pattern to that observed in adult-born GCs (Wallace et al., 2017). Similar to their morphological development, sensory activity regulates functional development in adult-born GL interneurons; odour enrichment accelerates the development of mature odour selectivity (Livneh et al., 2014).

## Conclusion

A clear picture is building of the major processes in OB development. For some of these processes (for example, M/TC dendritic pruning and synapse formation) this extends to some appreciation of the cellular and molecular mechanisms underlying particular maturational events. However, there is much we still do not know. Very little is understood regarding the developmental distinctions between different OB cell types and subtypes, not just the famously diverse GL interneurons, but also GC and projection neuron subclasses. We also require descriptions of *in vivo* sensory response properties of individual OB cell types during early postnatal development and longitudinal imaging of their morphological maturation. These immediate targets are all manifestly possible thanks to recent advances in single-cell -omics technology (Brann et al., 2020; Eng et al., 2019; Vickovic et al., 2019; Zeppilli et al., 2021) and neonatal live imaging (Fujimoto et al., 2019 preprint). The next big challenge will be to build on such descriptions of individual cell identity, morphology and function, to produce an integrated understanding of how developing OB neurons and glia interact in maturing circuits. OB development has an exciting future ahead!
